# AI-based radiodiagnosis using chest X-rays: A review

**DOI:** 10.3389/fdata.2023.1120989

**Published:** 2023-04-06

**Authors:** Yasmeena Akhter, Richa Singh, Mayank Vatsa

**Affiliations:** Indian Institute of Technology Jodhpur, Jodhpur, India

**Keywords:** chest X-ray, trusted AI, interpretable deep learning, Pneumoconiosis, tuberculosis, pneumonia, COVID-19

## Abstract

Chest Radiograph or Chest X-ray (CXR) is a common, fast, non-invasive, relatively cheap radiological examination method in medical sciences. CXRs can aid in diagnosing many lung ailments such as Pneumonia, Tuberculosis, Pneumoconiosis, COVID-19, and lung cancer. Apart from other radiological examinations, every year, 2 billion CXRs are performed worldwide. However, the availability of the workforce to handle this amount of workload in hospitals is cumbersome, particularly in developing and low-income nations. Recent advances in AI, particularly in computer vision, have drawn attention to solving challenging medical image analysis problems. Healthcare is one of the areas where AI/ML-based assistive screening/diagnostic aid can play a crucial part in social welfare. However, it faces multiple challenges, such as small sample space, data privacy, poor quality samples, adversarial attacks and most importantly, the model interpretability for reliability on machine intelligence. This paper provides a structured review of the CXR-based analysis for different tasks, lung diseases and, in particular, the challenges faced by AI/ML-based systems for diagnosis. Further, we provide an overview of existing datasets, evaluation metrics for different[][15mm][0mm]Q5 tasks and patents issued. We also present key challenges and open problems in this research domain.

## 1. Introduction

Advances in medical technology have enhanced the process of disease diagnosis, prevention, monitoring, treatment and care. Imaging technologies such as computer tomography (CT), medical imaging resonance (MRI), ultrasonography (USG), PET and others, along with digital pathology, are at ease for medical practitioners to assess and treat any disorder. Table 1 provides a comparative overview of the existing common imaging modalities used in medical sciences.^1^ ^2^ Every year across the globe, a massive number of investigations are performed to assess human health for disease diagnosis and treatment and the data generated from hospitals annually is in petabytes (IDC, 2014). The generated ‘big data' include all electronic health records (EHR) consisting of medical imaging, lab reports, genomics, clinical notes and financial and operational data (Murphy, 2019). Out of the total generated data from the hospital, the maximum contribution is made by radiology or imaging data. However, 97% of this data remained unanalyzed or unused (Murphy, 2019).

**Table 1:** Comparative analysis of common and widely used imaging modalities for medical applications.

**Specifications**	**CT**	**MRI**	**X-Ray**	**PET**	**SPECT**	**USG**
Acronynm	Computer Tomography	Magnetic Resonance Imaging	X-radiation/ Rontgen radiation	Positron Emission Tomography	Single Photon Emission Computed Tomography	Ultrasound/ Ultrasonography
Working principle	Uses multiple X-rays at different angles to generate 3D image	Uses magnet and pulsing radio waves to generate response from presence of water molecules inside the human body	X-ray beam passed through body gets blocked due to denser tissue which results in shadow of the tissue	Injection with Radioactive tracer that emits positrons. Later, these positrons are tracked over time in the form of a 3D image.	Same as PET	Uses high frequency sound waves as short pulses from area of interest as reflections received by transducer
Usage/ application	Recommended for all structures of human body (soft/ bone/blood vessels)	Best Suited for soft tissues	Recommended for diseased tissues/organs like lungs and bony structures such as teeth, skull etc.	Allows to trace the biological processes within human body	Same as PET	Best suited for internal organs. Not recommended for bony structures
Scanner cost ($)	85–450 K	225–500 K+	40–175 K	225–750 K	400–600 K	20–200 K
Radiation exposure	Yes	None	Yes	Yes	Yes	None
Per scan cost ($)	1,200–3,200	1,200–4,000	~70	3,000–6,000		100–1,000
Time of scanning	30 s	10 min–2 h	A few seconds	2–4 h	2–4 h	10–15 min
Side effect	Excessive exposure can lead to cancer		Prolonged exposure is hazardous	Radioactive allergy can occur. Overdue exposure can be dangerous	Same as PET	Comparatively safer
Spatial resolution (mm)	0.5–1	0.2	-	6–10	7–15	0.1–1
Details of soft/hard tissue	Higher contrast images are generated and ideal for both types of	Data with higher details of soft tissues are received	Can be used for soft tissues as well such Gall bladder, lungs etc.	Covers biological phenomenon such as drug delivery etc.	Allows to inspect functioning of various body organs and useful in brain disorders, heart problems and bone disorders	Soft tissues such as muscles, internal organs etc.
Limitations	Patients with large body size may underfit the scanning process	Patients with heavy weight may underfit the scanning process. Also, patients with pacemakers, tattoos are not advised the scan	Limited to few body parts	Kids and pregnant women are not recommended. Expensive.	Long scan time, low resolution, higher artifacts rate. Expensive	Objects deeper or hidden under bone are not captured. Presence of air spaces also fail scanning process.

Among all the imaging modalities, X-ray is the most common, fast and inexpensive modality used to diagnose many human body disorders such as fractures and dislocations and ailments such as cancer, osteoporosis of bones, chest conditions such as pneumonia, tuberculosis, COVID-19, and many more. It is a non-invasive and painless medical examination that uses an electric device for emission to pass through the patient body, and a 2-D image with the impression of internal body structures is generated. It is estimated that more than 3.5 billion diagnostic X-rays are performed annually worldwide (Mitchell, 2012) and they contribute 40% to the total imaging count per year (WHO, 2016), billion CXRs are performed worldwide. However, the availability of a trained workforce to handle this amount of workload is limited, particularly in developing and low-income nations. For instance, in some parts of India, there is one radiologist for 100,000 patients, and in the U.S., it is one radiologist for 10,000 patients.

In recent years, with the unprecedented advancements in deep learning and computer vision, computer-aided diagnosis has started to intervene in the diagnosis process and ease the workload for doctors. CXR-based analysis with machine learning and deep learning has drawn attention among researchers to provide an easy and reliable solution for different lung diseases. Many attempts have been made to provide easy automatic CXR-based diagnosis to increase the acceptance of AI-based solutions. Currently, many commercial products are available for clinical use which have cleared CE marked (Europe) and/or FDA clearance (United States), for instance, qXR by qure.ai (Singh et al., 2018), TIRESYA by Digitec (Kim et al., 2017), Lunit INSIGHT CXR by Lunit (Hwang et al., 2019), Auto lung by Samsung Healthcare (Sim et al., 2020), AI-Rad companion by Seimens Healthineers (Fischer et al., 2020), CAD4COVID-XRay by Thirona (Murphy et al., 2020) and many more.

**Figure 1:**
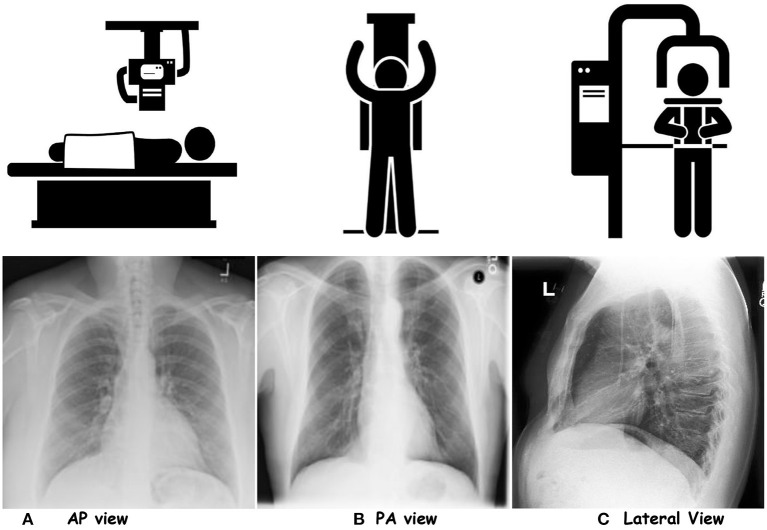
Showcasing the chest-X rays for three projections. **(A)** AP view, **(B)** PA view, and **(C)** Lateral View.

Based on the projection, CXRs are differentiated into three categories; posteroanterior (PA), anteroposterior (AP) and lateral (LL). Figure 1 showcases CXR samples for three different projections. PA view is the standard projection of the X-ray beam traversing the patient from posterior to anterior. On the other hand, AP is the opposite alternative to PA, where an X-ray beam passes the patient chest from anterior to posterior. A lateral view is performed erect left lateral (default). It demonstrates a better anatomical view of the heart, and assesses posterior costophrenic recesses. It is generally done to assess the retrosternal and retrocardiac airspaces.^3^ Table 2 tabulates the differences in the AP and PA views. The patient alignment also compromises the assessment of the chest X-ray for different organs such as the heart, mediastinum, tracheal position, and lung appearances. Rotation of the person can lead to certain misleading appearances in CXRs, such as heart size. In a left rotation in PA CXR, the heart appears enlarged and vice-versa. Moreover, the rotation can affect the assessment of soft tissue in CXRs, misleading the impressions in the lungs, for instance, costophrenic angle.^4^ About 25% of the total CXR count per year, faces the ‘reject rates' due to image quality or patient positioning (Little et al., 2017).

**Table 2:** Illustrates the differences between two common CXR projections.

**PA view**	**AP view**
Standard frontal Chest projection	Alternative frontal projection to the PA
X-ray beam traverses the patient from posterior to anterior	X-ray beam traverses the patient from anterior to posterior
Needs full aspiration and standing position from patient	Can be performed patient sitting on the bed
Best practice to examine lungs, mediastinum and thoracic cavity	Best practice for intubated and sick patients
Heart size appear normal	Heart size appear magnified
Images are of higher quality and a better option to assess heart size	Not a good option to assess the size of heart

**Figure 2:**
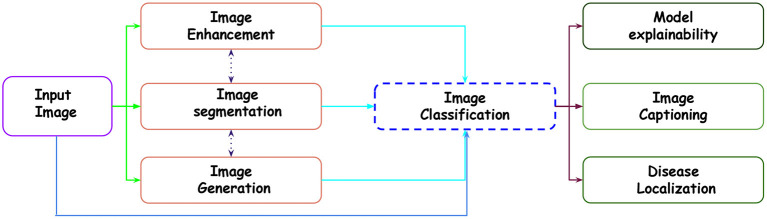
Showcasing the transition across different tasks in CXR-based analysis for a given input image.

In the existing literature, with the release of multiple datasets for different lung diseases, different tasks have been established with CXR data. Below is the list of tasks accomplished for CXR-based analysis using different ML and DL approaches. Figure 2 showcases the transition across different tasks for CXR-based image analysis.

- Image enhancement: The collected data from the hospitals do not always contribute to the detection process. The reason is varying quality samples. So, before proposing a detection pipeline, authors have used different CXR enhancement techniques for noise reduction, contrast enhancement, edge detection and many more.
- Segmentation: In CXR, segmentation of ROI usually gives a better edge to the disease detection pipeline. This reduces the ineffectual part of CXR, allowing lesser chances of misdiagnosis. Existing work has focused on the segmentation of the lung field, ribs, diseased part, diaphragm, costophrenic angle and support devices.
- Image classification: For the CXR datasets, multi-class and multi-label classification tasks have been performed using ML and DL approaches. With datasets such as CheXpert (Irvin et al., 2019), ChestXray14 (Wang et al., 2017) etc., multi-label classification is done. It reflects the different manifestations (local labels) in CXR due to any disease. For instance, Pneumoconiosis can cause multiple manifestations in the lung tissue, such as atelectasis, nodules, fibrosis, emphysema and many more. Similarly, in multi-class, we differentiate CXR into a particular class for diseases. For instance, the detection of pneumonia in CXR is a multi-class problem. We need to distinguish viral, bacterial and COVID-19, representing three classes (types) of pneumonia.
- Disease localization: It specifies the region within CXR infected by any particular disease. This is generally indicated by a bounding box, dot or circular shape.
- Image generation: Generally, the datasets are small in number and also suffer class imbalance problems. In order to improve the training set number, different approaches apart from affine transformation-based data augmentation, such as Generative Adversarial network-based approaches, are used. Moreover, analysis are done on the real and synthetic CXRs.
- Report generation: The generation of reports for a given CXR is one of the recent areas covered in CXR-based image analysis. The task involves reporting all the findings present in CXR in a text file.
- Model explainability: With the remarkable performance of the deep model, model explainability is a must to build trust in the machine intelligence-based decision. Explanation of machine intelligence justifies the decision process and builds trust in the automatic decision process. Interpretability encourages understanding the mechanism of algorithmic predictions.

**Figure 3:**
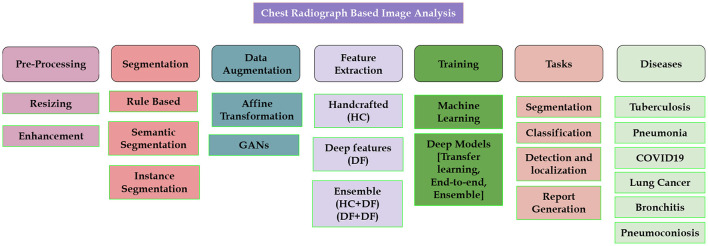
Showcasing research problems which have been studied in the literature.

The availability of intelligent machine diagnostics for Chest X-rays aids in reducing information overload and exhaustion of radiologists by interpreting and reporting the radiology scans. Many diseases affect the lungs, including lung cancer, bronchitis, COPD, Fibrosis, and many more. The literature review below is based on the publicly available datasets and the work done for these common diseases. Figure 3 showcases different areas for which existing literature is available for CXR-based analysis.

**Figure 4:**
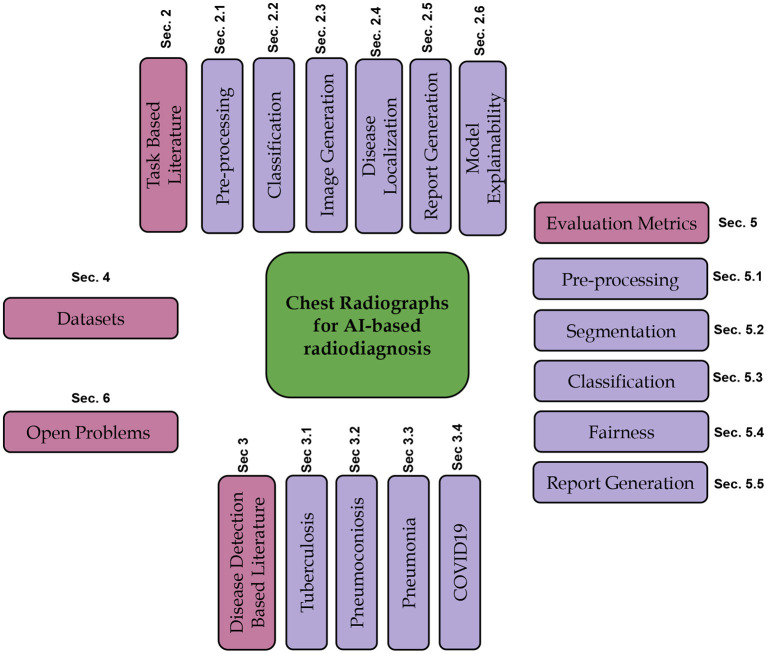
Illustrating the schematic structure of the paper.

In this review article, we focus on using computer vision, machine learning, and deep learning algorithms for different disorders where CXR is a standard medical investigation. We discuss the related work about the tasks mentioned above for CXR-based analysis. We further present the literature for widely studied disorders such as TB, Pneumonia, Pneumoconiosis, COVID-19, and lung cancer available in terms of publications and patents. We also discuss the evaluation metrics used to assess the performance of different tasks, publicly available datasets for various disorders and tasks. Figure 4 shows the schematic organization of the paper.

## 2. Task-based literature review

We first present the review of different tasks with respect to CXR-based analysis, such as pre-processing and classification and disease localization.

### 2.1. Image pre-processing

Image pre-processing includes enhancement and segmentation tasks and are either rule-based/handcrafted or deep learning based.

#### 2.1.1. Pre-deep learning based approaches

Sherrier and Johnson (1987) used a region-based histogram equalization technique to improve the image quality of CXR locally and finally obtain an enhanced image. Zhang D. et al. (2021) used the dynamic histogram enhancement technique (Abin et al., 2022) used different image enhancement techniques such as Brightness Preserving Bi Histogram (BBHE) (Zadbuke, 2012), Equal Area Dualistic Sub-Image Histogram Equalization (DSIHE) (Yao et al., 2016), Recursive Mean Separate Histogram Equalization (RMSHE) (Chen and Ramli, 2003) followed by a Particle swarm optimization (PSO) (Settles, 2005) for further enhancing the CXRs for detecting pneumonia. Soleymanpour et al. (2011) used adaptive contrast equalization for enhancement, morphological operation-based region growing to find lung contour for lung segmentation followed by oriental spatial Gabor filter (Gabor, 1946) for rib suppression. Candemir et al. (2013) used graph cut optimization (Boykov and Funka-Lea, 2006) method to find the lung boundary. Van Ginneken et al. (2006) used three approaches, Active shape model (Cootes et al., 1994), active appearance models (Cootes et al., 2001), pixelwise classification to segment the lung fields in CXRs. Li et al. (2001) used an edge detection-based approach by calculating vertical and horizontal derivatives to find the RoI in CXR. Annangi et al. (2010) used edge detection with an active contour method-based approach for lung segmentation.

#### 2.1.2. Deep learning based approaches

**Figure 5:**
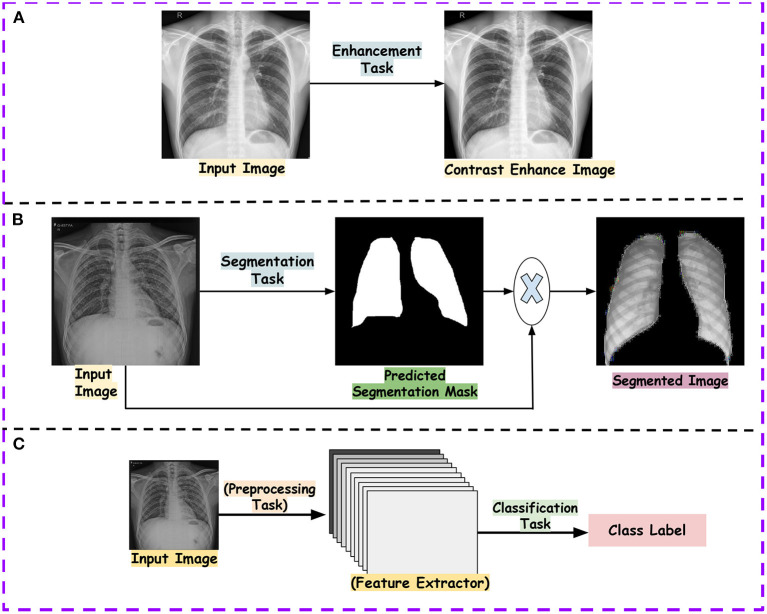
Showcases the examples of outputs obtained after tasks such as pre-processing and classification. **(A)** shows output of contrast enhancement. **(B)** shows output of the segmentation task and **(C)** shows the classification pipeline.

Abdullah-Al-Wadud et al. (2007) proposed enhancing the CXR images input for a CNN model for pneumonia detection. Hasegawa et al. (1994) used a shift-invariant CNN-based approach for lung segmentation. Hwang and Park (2017) proposed a Multi-stage training approach to perform segmentation using atrous convolutions. Hurt et al. (2020) used UNet-based (Ronneberger et al., 2015) semantic segmentation for extracting lung field and performed pneumonia classification. Li B. et al. (2019) used the UNet model to segment the lung part, followed by the attention-based CNN for pneumonia classification. Kusakunniran et al. (2021) and Blain et al. (2021) used UNet for lung segmentation for COVID-19 detection. Oh et al. (2020) used the extended fully convolution DenseNet (Jégou et al., 2017) to perform pixel-wise segmentation for lung fields in CXR to improve the classification performance for COVID-19 detection. Subramanian et al. (2019) used UNet based model to segment out the central venous catheters (CVCs) in CXRs. Cao and Zhao (2021) used a UNet-based semantic segmentation model with variational auto-encoder features in the encoder and decoder of UNet with an attention mechanism to perform automatic lung segmentation. Singh et al. (2021) propose an approach based on DeepLabV3+ (Chen et al., 2017b) with dilated convolution for lung field segmentation. Figures 5A, B showcase examples of the preprocessing tasks.

#### 2.1.3. Patent review

Hong et al. (2009a) proposed an approach to segment the diaphragm from the CXR using a rule-based method. Huo and Zhao (2014) proposed an approach to suppress the clavicle bone in CXR based on the edge detection algorithm. Chandalia and Gupta (2022) proposed a deep learning-based detection model to detect the inputted image as CT or CXR. Jiezhi et al. (2018) proposed a method to determine the quality of the inputted CXR image using deep learning.

#### 2.1.4. Discussion

From the above literature, pre-deep learning-based approaches require well-defined heuristics to either enhance or segment the lung region. A major focus has been laid on noise removal or contrast enhancement and lung segmentation. However, limited attention has been given to diseased ROIs segmentation. The common datasets used to perform lung segmentation are Montgomery and Shenzhen (Jaeger et al., 2014); however, the number of samples is limited. No dataset is available to focus on local findings.

### 2.2. Image classification

This section covers the literature on CXR classification for multiclass and multilabel settings. Input CXR images undergoes feature extraction followed by classification algorithms, which are either rule-based or handcrafted or use deep learning.

#### 2.2.1. Pre-deep learning based approaches

Katsuragawa et al. (1988) developed an automated approach based on the two-dimensional Fourier transform for detecting and characterizing interstitial lung disorder. The approach uses the textural information for a given CXR as normal or abnormal. Ashizawa et al. (1999) used 16 radiological features from CXR and ten clinical parameters to classify a given CXR as one of the classes among 11 interstitial lung diseases using ANN. A statistically significant improvement was reported over the diagnostic results from the radiologists.

#### 2.2.2. Deep Learning based approaches

Thian et al. (2021) combined two large publicly available datasets, ChestXray14 (Wang et al., 2017) and MIMICCXR (Johnson et al., 2019), to train a deep learning model for the detection of pneumothorax and assess its generalizability on six external validation CXR sets independent of the training set.

Homayounieh et al. (2021) proposed an approach to assess the ability of AI for nodule detection in CXR. The study included an in-house dataset trained on the deep model, which is pretrained on ChestXray14 (Wang et al., 2017) and ImageNet datasets for 14 class classifications. Lenga et al. (2020) used the existing continual learning approach for the medical domain for CXR-based analysis. Zech et al. (2018) assessed deep models for pneumonia using the training data from different institutions. Figure 5C showcases the classification pipeline using CXR for different lung diseases.

#### 2.2.3. Patent review

Lyman et al. (2019) proposed a model to differentiate CXR into normal or abnormal. The model is trained to find any abnormality like effusion, emphysema etc., to classify a given CXR as abnormal. Hong et al. (2009b) proposed a method for feature extraction to detect nodules in the CXR to reduce the false positives. Hong and Shen (2008) proposed an approach for automatically segmenting the heart region for nodule detection. Guendel et al. (2020) proposed a deep multitask learning approach to classify CXR for different findings present in it. The proposed approach also performs segmentation along with disease localization simultaneously. Clarke et al. (2022) proposed a computer-assisted diagnostic (CAD) method using wavelet transform-based feature extraction for automatically detecting nodules in the CXRs. Putha et al. (2022) proposed a deep learning-based method to predict the risk of lung cancer associated with the characteristics (size, calcification etc.) of nodules present in the CXR. Doi and Aoyama (2002) proposed a neural network-based approach to detect the presence of nodule and further classify them as benign or malignant. Lei et al. (2021) created a cloud-based platform for lung-based disease detection using CXR. Ting et al. (2021) proposed a transfer learning approach for detecting lung inflammation from a given CXR. Kang et al. (2019) proposed a transfer learning-based approach for predicting lung disease in the CXR image. Qiang et al. (2020) proposed a lung disease classification approach, which extracts the lung mask and enhances the segmented image and CNN-based model for feature extraction and classification. Luojie and Jinhua (2018) proposed a deep learning-based classification for lung disease for 14 different findings. Kai et al. (2019) proposed a deep learning system to classify the lung lesion in a given CXR. Harding et al. (2015) proposed an approach for lung segmentation and bone suppression in a given CXR to improve CAD results.

#### 2.2.4. Discussion

Researchers have generally developed algorithms for classification using supervised machine learning approaches. Both multilabel and multi-class classification tasks are studied. Due to availability of small sample size datasets with data imbalance, transfer learning is widely used in most research.

### 2.3. Image generation

**Figure 6:**
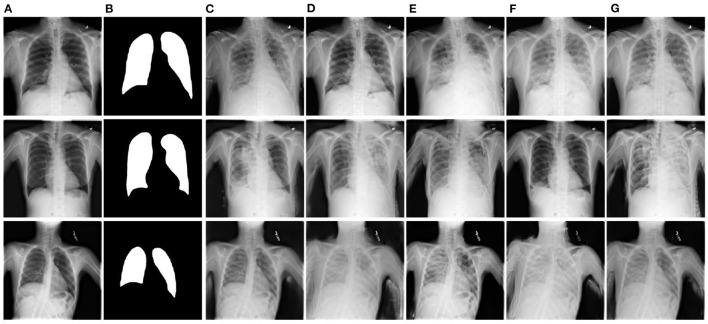
Showcasing the synthetically generated chest-X ray images. For a given normal image **(A)**, the proposed approach by Tang et al. (2019b) generates the abnormal images **(B–G)** is predicted segmentation mask for same input image and results in mask-image pairs from **(B–G)**. Figure is adapted from Tang et al. (2019b).

This section covers the existing work for the image generation task. This is a new field, where mostly generative models are used for other tasks and verify the model performance on synthetic and real CXR-based disease detection. Figure 6 showcases the synthetically generated CXR samples.

Tang et al. (2019b) proposed XLSor, a deep learning model for generating CXRs for data augmentation and a criss-cross attention-based segmentation approach. Eslami et al. (2020) proposed a multi-task GAN-based approach for image-to-image translation, generating bone-suppressed and segmented images using the JSRT dataset. Wang et al. (2018) proposed a hybrid CNN-based model for CXR classification and image reconstruction. Madani et al. (2018) used GAN based approach to generate and discriminate CXRs for the classification tasks. Sundaram and Hulkund (2021) used GAN based approach to perform data augmentation and evaluated the classification model for synthetically generated and affine transformation-based data in the CheXpert dataset.

#### 2.3.1. Discussion

The current work in image generation for CXR has focused on alleviating the data deficiency for training deep models. It is observed that the synthetic data generated using GAN-based approaches improve model performance compared to the standard data augmentation methods such as rotation and flip.

### 2.4. Disease localization

Disease localization is an interesting task for localizing diseased ROIs. This allows us to look at the difference between the predicted and the actual diseased area in the CXR. Yu et al. (2020) proposed a multitasking-based approach to segment Peripherally inserted central catheter (PICC) lines and detect tips simultaneously in CXRs. Zhang et al. (2019) proposed SDSLung, a multitasking-based approach adapted from Mask RCNN (Girshick et al., 2014) for lung field detection and segmentation. Wessel et al. (2019) proposed a Mask RCNN-based approach for rib detection and segmentation in CXRs. Schultheiss et al. (2020) used a RetinaNet (Ren et al., 2015) based approach to detect the nodule along with lung segmentation in CXRs. Kim et al. (2020) used Mask RCNN and RetinaNet to assess the effect of input size for nodule and mass detection in CXRs. Takemiya et al. (2019) proposed a CNN-based approach to perform nodule opacity classification and further used R-CNN to detect the nodules in CXRs. Kim et al. (2019) compared existing CNN-based object detection models for nodule and mass detection in CXRs. Cho et al. (2020) used a YOLO (Redmon and Farhadi, 2017) object detection model to detect different findings in CXRs.

#### 2.4.1. Patent review

Putha et al. (2021) proposed a deep learning-based system for detecting and localizing infectious diseases in CXR alongside using the information from the clinical sample for the same patient. Jinpeng et al. (2020) proposed a deep learning approach for automatic disease localization using CXRs based on weakly-supervised learning.

#### 2.4.2. Discussion

The current work in CXR-based analysis has focused on detecting the lung part in the given CXR or the disease area in the bounding box. Most of the work have used object detection algorithms such as YOLO, RCNN and its variants (Mask RCNN, Faster RCNN).

### 2.5. Report generation

This section covers the existing work in report generation for CXR image analysis. This is a recent area which combines two domains; Natural Language Processing (NLP) and Computer Vision (CV).

Xue et al. (2018) proposed a multimodal approach consisting of LSTM and CNN for the cohesive indent-based report generation with an attention mechanism. Li X. et al. (2019) proposed VisPi, a CNN and LSTM-based approach with attention to generating reports in medical imaging. The proposed algorithm performs classification and localization and then finally generates a detailed report. Syeda-Mahmood et al. (2020) proposed a novel approach to generate reports for fine-grained labels by fine-tuning the model learnt on fine-grained and coarse labels.

#### 2.5.1. Discussion

This recently explored area requires more attention. In CXR-based analysis, report generation allows a *multi-modal learning* using CNNs and sequential models. However, the task is challenging as the large text corpus is required with the CXR dataset and only a fewer datasets are available for this task.

### 2.6. Model explainability

Jang et al. (2020) trained a CNN on three CXR-based datasets (Asan Medical Center-Seoul National University Bundang Hospital (AMC-SNUBH), NIH, and CheXpert) for assessing the robustness of deep models in labeling noise. Authors added different noise levels in the labels of these datasets to demonstrate that the deep models are sensitive to the label noise; as for huge datasets, the labeling is done using report parsing or NLP, leading to a certain extent in labeling the CXR samples. Kaviani et al. (2022) and Li et al. (2021) reviewed different deep adversarial attacks and defenses on medical imaging. Li and Zhu (2020) proposed an unsupervised learning approach to detect the different adversarial attacks in CXRs and assess the robustness of deep models. Gongye et al. (2020) studied the effect of different existing adversarial attacks on the performance of the deep model for COVID-19 detection from CXRs. Hirano et al. (2021) studied the universal adversarial perturbations (UAP) effect on the deep model-based pneumonia detection and reported performance degradation in the classification of CXRs. Ma et al. (2021) studied the effect of altering the textural information present in the CXRs, which can lead to misdiagnosis. Seyyed-Kalantari et al. (2021) studied the fairness gaps in existing deep models and datasets for CXR classifications. Li et al. (2022) studied the gender bias affecting the performance of different deep models on existing datasets. Rajpurkar et al. (2017) used Class Activation Maps (CAMs) to interpret the model decisions for detecting different findings in CXRs. Pasa et al. (2019) used a 5-layered CNN-based architecture for detecting TB in CXRs from two publicly available datasets, Shenzhen and Montgomery. The authors used Grad-CAM visualization for model interpretability.

#### 2.6.1. Discussion

Work done so far on model interpretability for CXR-based disease detection is based on *post-hoc* approaches such as saliency map or CAM analysis. Explainability in AI-based decisions is a must to rely on machine intelligence. Healthcare is a challenging domain, and the life of humans is at risk based on a false positive or false negative. There is a need to incorporate the inbuilt model explainability to handle noisy or adversarial samples, thus improving model robustness for CXR-based systems. Further, challenges occur due to the data imbalance and less model interoperability, as models are usually trained on data from a single hospital. This results in unfair decisions by learning sensitive information from the data. The existing work should encourage more pathways for robust and fair CXR-based systems, which will further increase the chances of deployment of such systems in places with poor healthcare settings.

## 3. Disease detection based literature

**Figure 7:**
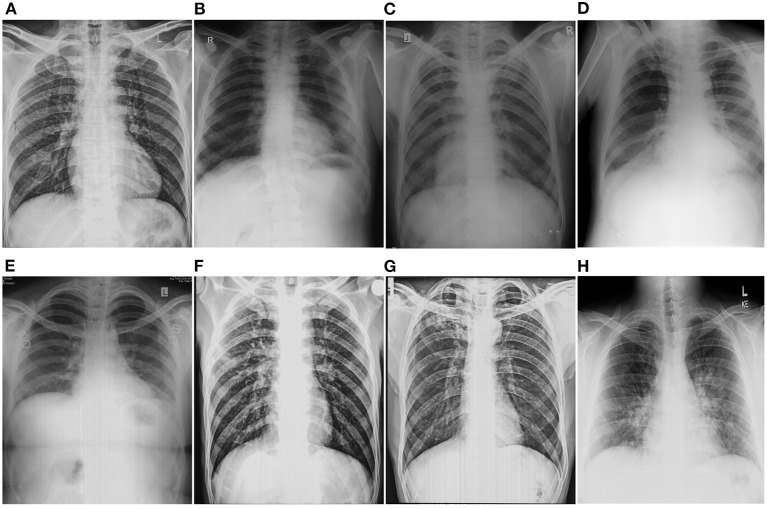
Showcasing the chest-X rays affected with different lung disorders. **(A)** Normal, **(B)** Pneumoconoisis, **(C)** TB, **(D)** Pneumonia, **(E)** Bronchitis, **(F)** COPD, **(G)** Fibrosis, and **(H)** COVID-19.

In this section, we present the literature review of commonly addressed lung diseases. Several CXR datasets are made publicly available, allowing to development of novel approaches for different disease-related tasks. Figure 7 showcases the samples of CXRs affected with different lung diseases.

### 3.1. Tuberculosis

TB is caused by *Mycobacterium tuberculosis*. It is one of the most common reasons for mortality in lung disease worldwide. About 10 million people were affected by TB in 2019 (WHO, 2021). In the year 2013, it took 1.5 million lives (WHO, 2013). TB is curable; however, hospital patient rush delays the diagnostic process and its treatment. CXR are the common radiological modality used to diagnose TB. Computer-aided diagnosis and CAD-based TB detection for CXR images will ease the detection process.

#### 3.1.1. Pre-deep learning based approaches

Govindarajan and Swaminathan (2021) used reaction-diffusion set method for lung segmentation followed by local feature descriptors such as Median Robust Extended Local Binary Patterns (Liu et al., 2016), local binary pattern (Liu et al., 2017) and Gradient Local Ternary Patterns (Ahmed and Hossain, 2013) with Extreme Learning Machine (ELM) and Online Sequential ELM (OSELM) (Liang et al., 2006) classifiers for detecting TB in CXR images using Montgomery dataset. Alfadhli et al. (2017) used speed-up robust features (SURF) (Bay et al., 2008) for feature detection and performed classification using SVM for TB diagnosis. Jaeger et al. (2014) collected different handcrafted features such as histogram of gradients (HOG) (Dalal and Triggs, 2005), the histogram of intensity, magnitude, shape and curvature descriptors, LBP Ojala et al., 1996) as set A for detection. They further used edge, color (fuzzy-color and color layout) based features as Set B for image retrieval. Chandra et al. (2020) used two-level hierarchical features (shape and texture) with SVM for TB classification. Santosh et al. (2016) used thoracic edge map encoding using PHOG (Opelt et al., 2006) for feature extraction followed by multilayer perceptron-based (MLP) based classification of CXR into TB or normal.

#### 3.1.2. Deep learning based approaches

Duong et al. (2021) created a dataset of 28,672 images by merging different publicly available datasets (Jaeger et al., 2014; Wang et al., 2017; Chowdhury et al., 2020; Cohen et al., 2020) for three class classification; TB, pneumonia and normal. Authors performed a deep learning-based classification using a pretrained EfficientNet (Tan and Le, 2019) trained on ImageNet (Deng et al., 2009) dataset, pretrained Vision Transformer (ViT) (Dosovitskiy et al., 2020) and finally developed a hybrid between EfficientNet and Vision Transformer. For the proposed hybrid model, the CXR is given input to the pretrained EfficientNet to generate features which are later fed to the ViT and finally, the classification results are obtained. Ayaz et al. (2021) proposed a feature ensemble-based approach for TB detection using Shenzen and Montgomery datasets. The authors used Gabor filter-based handcrafted features and seven different deep learning architectures to generate the deep features. Dasanayaka and Dissanayake (2021) proposed a deep learning-algorithm comprising of data generation using DCGAN (Radford et al., 2015), lung segmentation using UNet (Ronneberger et al., 2015) and transfer learning approach based feature ensemble and classification. The authors used genetic algorithm-based hyperparameter tuning. Msonda et al. (2020) used the deep model-based approach with spatial pyramid pooling and analyzed its effect on TB detection using CXR allowing robustness to the combination of features, thus improving the performance. Sahlol et al. (2020) proposed an Artificial Ecosystem-based Optimization (AEO) (Zhao et al., 2020) on top of the features extracted from a pre-trained network, MobileNet, trained on ImageNet dataset as feature selector. The authors used two publicly available datasets, Shenzen and Pediatric Pneumonia CXR dataset (Kermany et al., 2018b). Rahman et al. (2020b) used a deep learning approach for CXR segmentation and classification into TB or normal. For segmentation, the authors used two deep models, UNet and modified UNet (Azad et al., 2019). Authors also used different existing visualizations techniques such as SmoothGrad (Smilkov et al., 2017), Grad-CAM (Selvaraju et al., 2017), Grad-CAM++ (Chattopadhay et al., 2018), and Score-CAM (Wang H. et al., 2020) for interpreting deep model for making classification decisions. The authors used nine different deep models for CNN-based classification of CXR into TB or normal. Rajaraman and Antani (2020) created three different models for three different lung diseases. First model was trained and tested on RSNA pneumonia (Stein et al., 2018), pediatric pneumonia (Kermany et al., 2018b), and Indiana (McDonald et al., 2005) datasets for pneumonia detection. The second model is trained and tested for TB detection using the Shenzhen dataset. Finally, the first model is finetuned for TB detection to improve model adaption for a new task and reported majority voting results for TB classification. Rajpurkar et al. (2020) collected CXRs from HIV-infected patients from two hospitals in South Africa and developed CheXaid, a deep learning algorithm for the detection of TB to assist clinicians with web-based diagnosis. The proposed model consists of DenseNet121 trained on CheXpert (Irvin et al., 2019) dataset, and outputs six findings (micronodular, nodularity, pleural effusion, cavitation, and ground-glass) with the presence or absence of TB in a given CXR. Zhang et al. (2020) proposed an attention-based CNN model, CBAM, and used channel and spatial attention to generate more focus on the manifestation present in the TB CXR. The authors used different deep models and analyzed the effect of the attention network on detecting TB. Table 3 summarizes the above work for TB detection using CXRs.

**Table 3:** Review of the literature for TB detection using CXRs based on different feature extraction methods.

**References**	**Highlights**	**Pretraining**	**Dataset**
Govindarajan and Swaminathan (2021)	Texture-based feature descriptors with ML classifier	No	Montgomery
Alfadhli et al. (2017)	Used SURF as feature extractor and SVM as classifier	No	Montgomery
Jaeger et al. (2014)	Used texture-based features (LBP, HOG) and statistical feature with ML Classifier	No	Shenzhen, Montgomery
Chandra et al. (2020)	Used shape and textural features with SVM	No	Shenzhen, Montgomery
Santosh et al. (2016)	Used PHOG as features with MLP as classifier	No	Shenzhen, Montgomery
Duong et al. (2021)	Used Pretrained EfficientNet and ViT, and developed a hybrid of two	Yes	Shenzhen, Montgomery, Chestxray14, COVID-CXR (Chowdhury et al., 2020)
Ayaz et al. (2021)	Used Feature ensemble of handcrafted and deep features	Yes	Shenzen, Montgomery
Dasanayaka and Dissanayake (2021)	Generated synthetic images, performed segmentation and used feature ensemble for classification	Yes	Shenzhen, Montgomery
Msonda et al. (2020)	Used spatial pyramid pooling for deep feature extraction	Yes	Shenzhen, Montgomery, private
Sahlol et al. (2020)	Used Meta-heuristic approach for Deep feature selection	Yes	Shenzen, Montgomery, PedPneumonia
Rahman et al. (2020b)	Performed segmentation and used different visualization techniques	Yes	Shenzhen, Montgomery, NIAID TB, RSNA
Rajaraman and Antani (2020)	Performed tri-level classification and studied task adaptation	Yes	RSNA pneumonia, PedPneumonia, Indiana, Shenzhen
Rajpurkar et al. (2020)	Developed a web-based system for TB affected HIV patients	Yes	CheXpert private dataset
Zhang et al. (2020)	Used deep model with Attention based CNN (CBAM) module	Yes	Shenzhen, Montgomery
Rahman M. et al. (2021)	Merged publicly available CXR dataset with XGBoost as classifier	Yes	Shenzhen, Montgomery
Owais et al. (2020)	Used a feature ensemble by combining low and high level features	Yes	Shenzhen, Montgomery
Das et al. (2021)	Modified a pre-trained InceptionV3 for TB classification	Yes	Shenzhen, Montgomery
Munadi et al. (2020)	Used enhancement techniques to improve deep classification	Yes	Shenzhen, Montgomery
Oloko-Oba and Viriri (2020)	Used deep learning-based pipeline for classification	Yes	Montgomery
Ul Abideen et al. (2020)	Proposed the Bayesian CNN to deal with uncertain TB and non-TB cases that have low discernibility.	Yes	Shenzhen, Montgomery
Hwang et al. (2016)	Proposed a modified AlexNet-based model for end-to-end training. Also performed cross-database evaluations.	Yes	Shenzhen, Montgomery
Gozes and Greenspan (2019)	Proposed MetaChexNet, trained on CXRs and metadata of gender, age and patient positioning. Later, finetuned the model for TB classification	Yes	ChestXray14, Shenzhen, Montgomery

Pretraining (yes/no) refers to the use of weights of a deep model trained on ImageNet dataset. Private refers that the data used being in-house and is not released publicly.

#### 3.1.3. Patent review

Kaijin (2019) proposed a deep learning-based approach for segmentation and pulmonary TB detection in CXR images. Venkata Hari (2022) proposed a deep learning model for detecting TB in chest X-ray images. Chang-soo (2021) proposed an automatic chest X-ray image reader which involves reading data from the imaging device, segments the lung part, followed by gray level co-occurrence matrix-based feature extraction and finally discriminates it as normal, abnormal or TB. Minhwa et al. (2017) proposed a CAD-based system for diagnosing and predicting TB in CXR using deep learning.

#### 3.1.4. Discussion

In most handcrafted approaches, the texture of CXR is used to define features, followed by any ML classifier. From the above, it is highlighted that the major focus for TB detection is on two datasets; Shenzhen and Montgomery. However, the two datasets contain below 1000 samples even when combined together. This results in poor generalization and needs a pretrained backbone network which is finetuned later. This is why pretrained models trained on the ImageNet dataset are widely used for TB classification from CXRs. Thus, there is a need for large-scale datasets for TB detection with segmentation masks and disease annotations to achieve model generalizability and interpretability.

### 3.2. Pneumoconoisis

Pneumoconoisis is a broad term that describes lung diseases among industry workers due to overexposure to silica, coal, asbestos, and mixed dust. It is an irreversible and progressive occupational disorder prevalent worldwide and is becoming a major cause of death among workers. It is further categorized based on elements inhaled by the workers, such as silicosis (silica), brown lung (cotton and other fiber), pneumonoultramicroscopicsilicovolcanoconiosis (ash and dust), coal worker Pneumoconiosis (CWP) or black lung (asbestos), and popcorn lung (Diacetyl). People exposed to these substances are at a high risk of developing other lung diseases such as lung cancer, lung collapse, and TB.

#### 3.2.1. Pre-deep learning based approaches

Okumura et al. (2011) proposed a rule-based model for detecting the region of interests (ROIs) for nodule patterns based on the Fourier transform and an ANN-based approach for other ROIs which were not covered using the power spectrum analysis. The dataset is based on 11 normal and 12 abnormal cases of Pneumoconiosis, where normal cases were selected from an image database of the Japanese Society of Radiological Technology. Abnormal cases were selected randomly from the digital image database. Ledley et al. (1975) demonstrated the significance of textural information present in the CXR to detect the presence of coal work Pneumoconiosis (CWP). Hall et al. (1975) used the textural information present in CXRs and generated features based on spatial and histogram moments for six regions of a given segmented image. Authors performed classification based on maximum likelihood estimation and linear discriminant analysis (LDA). The authors further performed 4 class profusion classification for a given CXR in CWP workers. Yu et al. (2011) used the active shape modeling to segment out the lung from the CXR. The segmented image is divided into six non-overlapping zones as per the ILO guidance. On top of this, six separate SVM classifiers are built on the histogram and co-occurrence-based features generated from each zone. The authors also generated a chest-level classification by integrating the prediction results of the six regions. The experiments are carried out on a dataset of 850 PA CXRs with 600 normal and 250 abnormal cases collected from Shanghai Pulmonary Hospital, China. Murray et al. (2009) proposed based on an amplitude-modulation frequency-modulation (AM-FM) approach to extract the features and used partial least squares for classification. The authors extracted AM-FM features for multiple scales and used a classifier for each scale, later combining results from the individual classifiers. The authors performed the experiments on the CXRs collected from the Miners' Colfax Medical Center and the Grant's Uranium Miners, Raton, New Mexico, for CWP detection. Xu et al. (2010) collected a private dataset of 427 CXR images, consisting of 252 and 175 images for normal and Pneumoconiosis, respectively. The authors performed segmentation using an active shape model followed by dividing the image into six sub-regions. For each subregion, five co-occurrence-based features are extracted. A separate SVM is trained for each subregion, followed by the staging of Pneumoconiosis using a separate SVM.

#### 3.2.2. Deep learning based approach

Yang et al. (2021) proposed a deep learning-based approach for Pneumoconiosis detection. The proposed approach consists of a two-stage pipeline, UNet (Ronneberger et al., 2015) for lung segmentation and pre-trained ResNet34 for feature extraction on the segmented image. The dataset is collected in-house and includes 1,760 CXR images for two classes; normal and Pneumoconiosis. Zhang L. et al. (2021) proposed a deep model for screening and staging Pneumoconiosis by dividing the given CXR into six subregions. This was followed by a CNN-based approach to detect the level of opacity in each subregion, and finally, a 4-class classification was performed to determine normal I, II, and III stages of Pneumoconiosis for a UNet-based segmented image. The results are obtained on the in-house data of 805 and 411 subjects for training and testing, respectively. Devnath et al. (2021) applied a deep transfer learning CheXNet (Rajpurkar et al., 2017) model on a private dataset. The approach is based on the multilevel features extracted from the CheXNet and fed to a different configuration of SVMs. Wang X. et al. (2020) collected a dataset of 1881, including the 923 and 958 samples for Pneumoconiosis and normal, respectively. They used InceptionV3, a deep learning architecture to detect Pneumoconiosis in the given CXR to determine the potential of deep learning for assessing Pneumoconiosis. Wang D. et al. (2020) generated synthetic data for both normal and Pneumoconiosis using CycleGAN (Zhu et al., 2017), followed by a CNN-based classifier. The author proposed a cascaded framework of pixel classifier for lung segmentation, CycleGAN, for generating training images and a CNN-based classifier. Wang et al. (2021) collected a set of in-house 5,424 CXRs, including normal and Pneumoconiosis cases, belonging to 4 different stages (0–3). Authors used ResNet101 (He et al., 2016) for detecting Pneumoconiosis on segmented images from the UNet segmentation model and showed improved results compared to radiologists. Sydney, and Wesley Medical Imaging, Queensland, Australia. Hao et al. (2021) collected data consisting of 706 images from Chongqing CDC, China, with 142 images positive for Pneumoconiosis. Authors trained two deep learning architectures, ResNet34 and DenseNet53 (Huang et al., 2017) for the classification of CXRs into normal or Pneumoconiosis. Table 4 summarizes the above work based on the method of feature extraction.

**Table 4:** Review of the literature for Pneumoconiosis detection using CXRs.

**References**	**Highlights**	**Pretraining**	**Dataset**
Okumura et al. (2011)	Used Fourier Transform to demonstrate the nodule pattern with Neural Nets for detection &	No	JSRT, Private
Hall et al. (1975)	Used textural for six regions to determine profusion level	No	Private
Yu et al. (2011)	Used active shape model to segment lung, divided each lung into six regions. Features generated from each region are used to train SVM	No	Private
Xu et al. (2010)	Used textural features generated from six lung regions with SVM for classification and staging	No	Private
Yang et al. (2021)	Two stage pipeline with segmentation followed by classification	Yes	Private
Zhang L. et al. (2021)	Used Deep learning for screening and staging based on six lung regions	Yes	Private
Devnath et al. (2021)	Used Feature ensemble of multiple level deep features generated from pretrained model on CXR data	Yes	ChestXray14, private
Wang X. et al. (2020)	Used InceptionNet for end-to-end classification	No	Private
Wang D. et al. (2020)	Generated synthetic CXR samples and trained CNN with real and synthetic	Yes	Chestxray14, Private
Wang et al. (2021)	Performing Pneumoconioisis staging on segmented CXR images	Yes	Private
Hao et al. (2021)	Used two different deep models with different depths for feature generation, followed by classification	Yes	Private

Pretraining (yes/no) refers to the use of weights of deep models trained on the ImageNet dataset. Private refers that the data used being in-house and is not released publicly.

#### 3.2.3. Patent review

Sahadevan (2002) proposed an approach to use high-resolution digital CXR images to detect early-stage lung cancer, Pneumoconiosis and pulmonary diseases. Wanli et al. (2021) proposed a deep learning-based approach for Pneumoconoisis detection using lung CXR image.

#### 3.2.4. Discussion

From the above-cited work, it is clear that there is no publicly available dataset. The current work is done on the in-house datasets with fewer samples. This draws our attention to the fact that the automatic detection of Pneumoconiosis from CXRs requires publicly available datasets for developing robust, generalizable and efficient algorithms.

### 3.3. Pneumonia

It is a viral or bacterial infection affecting the lungs and humans of all ages, including children. CXRs are widely used to examine the manifestation caused due to pneumonia infection.

Sousa et al. (2014) compared different machine learning models for the classification of pediatric CXRs into normal or pneumonia. Zhao et al. (2019) merged four different CXR datasets for pneumonia classification and performed lung and thoracic cavity segmentation using DeepLabv2 (Chen et al., 2017a) and ResNet50 for pneumonia classification from CXRs on top of the segmented images. Tang et al. (2019a) used CycleGAN to generate synthetic data and proposed TUNA-Net to adapt adult to pediatric pneumonia classification from CXRs. Narayanan et al. (2020) used UNet for lung segmentation followed by a two-level classification viz; level 1 classifies given CXR into pneumonia or normal, and level 2 further classifies pneumonia CXR into either bacterial or viral class. Rajaraman et al. (2019) highlighted different visualization techniques for interpreting CNN-based pneumonia detection using CXRs. Ferreira et al. (2020) used VGG16 for classifying pediatric CXR into normal pneumonia and further classifying them as bacterial or viral. Zhang J. et al. (2021) proposed an EfficientNet-based confidence-aware anomaly detection model to differentiate viral pneumonia as a one-class classification from non-viral and normal classes (Elshennawy and Ibrahim, 2020; Longjiang et al., 2020; Yue et al., 2020) used different deep learning models using a transfer learning approach to perform classification using CXRs for pneumonia. Mittal et al. (2020) used an ensemble of CNN and CapsuleNet (Sabour et al., 2017) for detecting pneumonia from CXRs images using publicly available pediatric dataset (Stein et al., 2018). Rajpurkar et al. (2017) proposed a pre-trained DenseNet121 model for classifying 14 findings present in CXRs in Chestxray14 dataset. The authors further performed a binary classification to detect pneumonia. Table 5 summarizes the above based on the feature extraction methods.

**Table 5:** Summarizes the literature for Pneumonia detection using CXR.

**References**	**Highlights**	**Pretraining**	**Dataset**
Sousa et al. (2014)	Compared different ML classifiers for Pediatric Pneumonia	No	PedPneumonia
Zhao et al. (2019)	Used Multiple datasets and performed semantic lung segmentation	No	PedPneumonia, RSNA-Pneumonia, Private
Tang et al. (2019a)	Generated synthetic data and trained model for adult pneumonia, and later adapted that for pediatric pneumonia	No	RSNA, PedPneumonia
Narayanan et al. (2020)	Lung segmentation followed by two level of classification	Yes	PedPneumonia
Rajaraman et al. (2019)	Comparison of different visualization techniques for deep model explaination	Yes	PedPneumonia
Ferreira et al. (2020)	A multistage CXR classification viz; healthy or pneumonia and viral or bacterial pneumonia	Yes	PedPneumonia
Zhang J. et al. (2021)	EfficientNet-based confidence-aware anomaly detection model	No	PedPneumonia
Mittal et al. (2020)	Used an ensemble of deep model (CNN) and CapsuleNet	Yes	PedPneumonia
Rajpurkar et al. (2017)	Performed multilabel classification with CAM analysis	Yes	Chestxray14

Pretraining (yes/no) refers to the use of weights of a deep model trained on the ImageNet dataset. Private refers that the data used is in-house and is not released publicly.

#### 3.3.1. Patent review

Shaoliang et al. (2020) proposed a system for pneumonia detection from CXR using deep learning based on transfer learning.

#### 3.3.2. Discussion

Most of the work is done around (Stein et al., 2018) dataset in multi-class settings. However, there are challenges which need to be addressed other than the dataset challenge, which includes lung segmentation and model interpretability. Transfer learning is widely used to improve generalization for Pneumonia detection on CXRs. Pneumonia is a common manifestation of many lung disorders and is thus required to be detected in multilabel settings.

### 3.4. COVID-19

COVID-19 is caused due to SARS-CoV-2 Coronavirus prevalent worldwide and is responsible for the ongoing pandemic. It is responsible for the death of more than 6 million people worldwide. Rt-PCR is an available test to detect the presence of COVID-19; however, using CXR is a rapid method for diagnosis and detecting the presence of pneumonia-like symptoms in the lungs.

#### 3.4.1. Pre-deep learning based approaches

Rajagopal (2021) used both transfer learning (pre-trained VGG16) and ML (SVM, XGBoost) trained on a deep features-based approach for three-class classification for COVID-19 detection. Jin et al. (2021) used a pre-trained AlexNet to generate the features on CXR images followed by feature selection and classification using SVM.

#### 3.4.2. Deep learning based approaches

Chowdhury et al. (2020) proposed a dataset by merging publicly available datasets (Wang et al., 2017; Mooney, 2018; Cohen et al., 2020; ISMIR, 2020; Rahman et al., 2020a; Wang L. et al., 2020) for COVID-19 and used eight pretrained CNN models [MobileNetv2, SqueezeNet, ResNet18, ResNet101, DenseNet201, Inceptionv3, ResNet101, CheXNet (Rajpurkar et al., 2017), and VGG19] for the three class classification; normal, viral pneumonia, and COVID-19 pneumonia. Khan et al. (2020) proposed CoroNet, a transfer learning-based approach using XceptionNet-based approach, trained end-to-end for classification of CXR images into normal, bacterial pneumonia, viral pneumonia, COVID-19 using publicly available datasets. Islam et al. (2020) proposed a CNN-LSTM based architecture for detecting COVID-19 from CXRs for a dataset of 4,575 images. Pham (2021) compared the fine-tuning approach with the recently developed deep architectures for 2-class and 3-class classification problems for COVID-19 detection in CXRs on three publicly available datasets. Al-Rakhami et al. (2021) extracted deep features from pre-trained models and performed classification using RNN. Duran-Lopez et al. (2020) proposed COVID-XNET, for detecting COVID-19 from CXR images based on CNN for binary classification. Gupta et al. (2021) proposed InstaCovNet-19, by stacking different fine-tuned deep models with variable depth as to increase model robustness for COVID-19 classification on CXRs. Punn and Agarwal (2021), Wang N. et al. (2020), Khasawneh et al. (2021), Jain et al. (2021), El Gannour et al. (2020), Panwar et al. (2020b), and Panwar et al. (2020a) used transfer learning based approach for differentiating COVID-19 from viral pneumonia and normal CXRs. Abbas (2021) proposed a CNN-based class decomposition approach, DeTraC, which aims to decompose classes into subclasses and assign new labels independent of each other within the datasets by adding a class decomposition layer and later adding back these subsets to generate final predictions. The authors used the COVID-19 Classification from CXR images on publicly available datasets. Gour and Jain (2020) proposed a stacked CNN-based approach using five different submodules from two different deep models; first fine-tuned VGG16 and second a 30-layered CNN, and the output is combined by logistic regression for three classifications for COVID-19 using CXRs. Malhotra et al. (2022) proposed COMiT-Net, a deep learning-based multitasking approach for COVID-19 detection from CXR, simultaneously performs semantic lung segmentation, and disease localization to improve model interpretability. Pereira et al. (2020) combined both handcrafted and deep learning-based features and performed two-level classification for COVID-19 detection. Rahman T. et al. (2021) compared the effect of different enhancement techniques and lung segmentation on classification tasks based on transfer learning for differentiating CXRs as COVID-19, normal, and Non-COVID. Li et al. (2020) developed COVID-MobileXpert, a knowledge distillation-based approach consisting of three models, one large teacher model, trained on a large CXR dataset and two student models; one finetuned on COVID-19 dataset to discriminate COVID-19 pneumonia from normal CXRs and another a small lightweight model to perform on-device screening for CXR snapshots. Ucar and Korkmaz (2020) proposed Bayes-Squeeznet, based on pretrained SqueezeNet and Bayesian optimization for COVID-19 detection in CXRs. Shi et al. (2021) proposed a knowledge distillation-based attention method with transfer learning for COVID-19 detection from CT and CXRs. Saha et al. (2021) proposed EMCNet, based on extracting deep features from CXRs and training different machine learning classifiers. Mahmud et al. (2020) proposed a CovXNet, based on training a deep model on different resolution CXR data, Stacked CovXNet, and later finetune it on COVID-19 and non-COVID-19 CXR data as a target task. Table 6 summarizes the above work for COVID-19 detection using CXRs.

**Table 6:** Review of the literature for COVID19 detection using CXRs.

**References**	**Highlights**	**Pretraining**	**Dataset**
Rajagopal (2021)	Combined deep learning and ML classifier	Yes	PedPneumonia, COVID-CXR, https://github.com/agchung
Jin et al. (2021)	Used deep feature followed by feature selection with SVM	Yes	PedPneumonia, COVID-CXR
Chowdhury et al. (2020)	Used deep ensemble feature generation	Yes	Mutiple datasets with different disorders
Khan et al. (2020)	XceptionNet based end-to-end training	Yes	PedPneumonia, COVID-CXR, COVIDDGR
Islam et al. (2020)	Used a combination of LSTM-CNN-based architecture	Yes	Combination of
publicly available data Pham (2021)	Used a multi-level classification approach for two and three disease classes	Yes	COVID-CXR, PedPneumonia, COVID-19 (kaggle), ActualMed (github)
Al-Rakhami et al. (2021)	Approach combines CNNs with sequential deep model	Yes	Data collected from various available sources
Duran-Lopez et al. (2020)	Proposed COVID-XNet, a custom deep learning model for binary classification	Yes	BIMVC, COVID-CXR
Gupta et al. (2021)	Proposed InstaCovNet-19, with ensemble generated from deep features	Yes	Chowdhury et al. (2020), COVID-CXR
Abbas (2021)	Class decomposition into sub-classes with pre-trained models	Yes	JSRT, COVID-CXR
Gour and Jain (2020)	Submodule stacking from pretrained and customized deep models	Yes	COVID-CXR, ActualMed, PedPneumonia
Malhotra et al. (2022)	Multi-task approach with segmentation, disease classification and	Yes	CheXpert, Chestxray14, BIMVC-COVID19, Various online sources
Pereira et al. (2020)	Feature ensemble of handcrafted and deep features	Yes	COVID-CXR, Chestxray14, Radiopedia Encyclopedia
Rahman T. et al. (2021)	Employed and compared different enhancement technique for performance improvement	Yes	PedPneumonia, BIMCV+COVID19
Li et al. (2020)	On-device detection approach for CXR snapshots	Yes	PedPneumonia, COVID-CXR
Ucar and Korkmaz (2020)	Used Bayesian optimization with deep models for differentiating Pneumonia	Yes	PedPneumonia, COVID-CXR
Shi et al. (2021)	Knowledge transfer in the form of attention from teacher to student network	No	COVID-CXR, SIRM
Saha et al. (2021)	Used deep features with different ML classifiers	Yes	COVID-CXR, SIRM, PedPneumonia, Chestxray14,
Mahmud et al. (2020)	Used feature stacking generated from different resolutions	Yes	PedPneumonia, private

Pretraining (yes/no) refers to use of weights of deep model trained on ImageNet dataset. Private refers that the data used is in-house and is not released publicly.

#### 3.4.3. Patent review

Shankar et al. (2022) proposed a deep learning-based SVM approach for classifying chest X-rays affected with COVID-19 or normal.

#### 3.4.4. Discussion

The research is very recent, and papers produced on different datasets are generated either with fewer samples or combining more than one dataset. The CXR data released post-pandemic is collected from multiple centers across the globe. Further, only a fewer works have incorporated inherent model interpretability. To the best of our knowledge, no work has been established for segmentation, report generation, or disease localization and the primary focus is on the classification task.

## 4. Datasets

**Figure 8:**
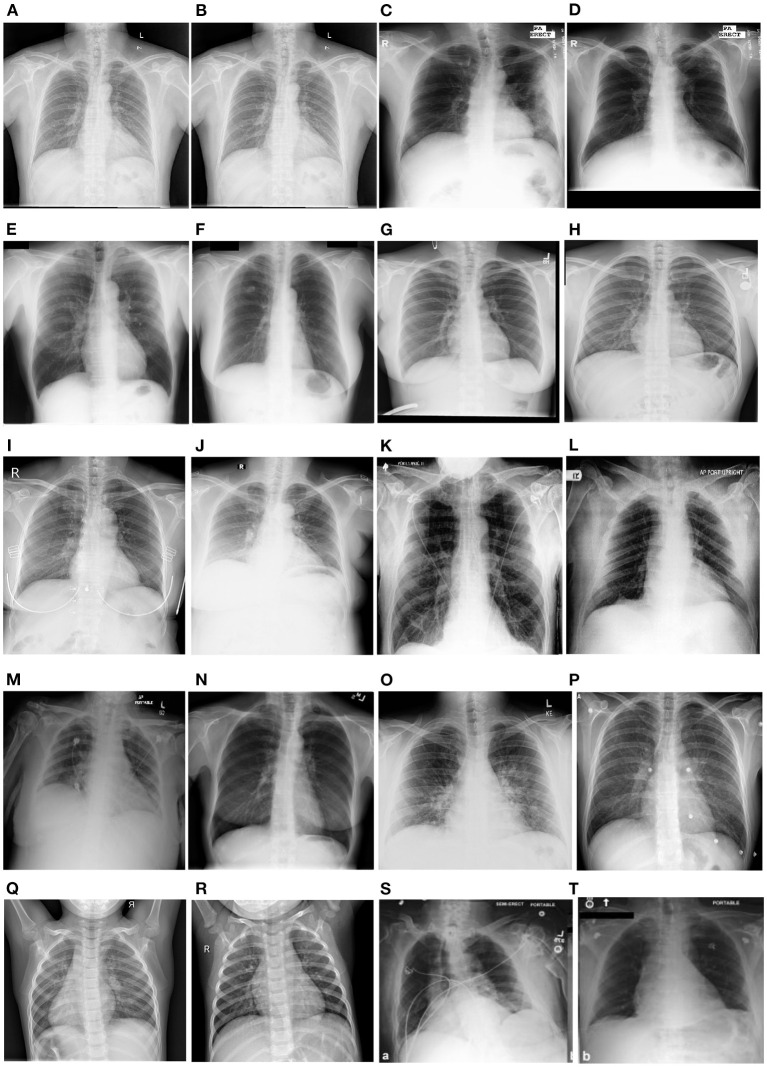
Showcases the sample examples of the CXRs from different datasets. The samples belong to Shenzhen **(A, B)**, Montgomery **(C, D)**, JSRT **(E, F)**, Chestxray14 **(G, H)**, VinDr-CXR **(I, J)**, CheXpert **(K, L)**, RSNA Pneumonia **(M, N)**, Covid-CXR **(O, P)**, PedPneumonia **(Q, R)**, and MIMIC-CXR **(S, T)**. The samples across different datasets highlight a wide variety in terms of quality, contrast, brightness and original image size.

Several chest X-ray datasets have been released over the past. These datasets are either made available in DICOM, PNG or JPEG format. The labeling is either done with the help of experts in this domain or label extraction methods using the natural language processing techniques from the reports associated with each image. Moreover, a few datasets also include the local labels as disease annotations for a given sample. Authors have also included lung field masks available as ground truth for performing segmentation and associated tasks. In this section, we include the publicly available datasets used in the literature. The statistics are also summarized in Table 7. Figure 8 illustrates the samples from the different CXR datasets mentioned below.

- JSRT: Shiraishi et al. (2000) introduced the dataset in the year 2000, consisting of 247 images for two classes; malignant and benign. The resolution of each image is 2048 X 2048. The dataset can be downloaded from http://db.jsrt.or.jp/eng.php.
- Open-i (O) : Demner-Fushman et al. (2012) proposed the chest X-ray dataset consisting of 3955 samples for 3955 subjects. Images are available in DICOM format. The findings are available in the form of reports made available by the radiologists. The dataset is collected from Indiana Network for Patient care (McDonald et al., 2005). The dataset can be downloaded from https://openi.nlm.nih.gov/.
- NLST : The dataset available collected from the NLST screening trails (Team, 2011). The dataset consists of 26,732 subjects for CXRs, and a subset of the dataset is available on request from https://biometry.nci.nih.gov/cdas/learn/nlst/images/.
- Shenzhen: Jaeger et al. (2014) introduced the dataset in the year 2014, consisting of 662 CXRs belonging to two classes; Normal and TB. The dataset was collected from Shenzhen No.3 Hospital in Shenzhen, Guangdong providence, China, in September 2012. The samples are shared publicly with original full resolution and include lung segmentation masks. The dataset can be downloaded from https://openi.nlm.nih.gov/imgs/collections/ChinaSet_AllFiles.zip.
- Montgomery: Jaeger et al. (2014) introduced the dataset in the year 2014, consisting of 138 CXRs belonging to two classes; Normal and TB. The dataset is collected from the tuberculosis control program of the Department of Health and Human Services of Montgomery County, MD, USA. It also includes lung segmentation masks, which are shared as original full-resolution images. The dataset can be downloaded from https://openi.nlm.nih.gov/imgs/collections/NLM-MontgomeryCXRSet.zip.
- KIT: Ryoo and Kim (2014) proposed the dataset in year 2014. It consists of 10,848 DICOM CXRs with 7020 for normal and 3828 for TB. The dataset is collected from the Korean Institute of TB.
- Indiana: Demner-Fushman et al. (2016) introduced the dataset in year 2015. The dataset is collected from the Indiana University hospital network. The dataset includes 3996 radiology reports and 8121 associated images. The dataset can be downloaded from https://openi.nlm.nih.gov/.
- Chestxray8: Wang et al. (2017) released the dataset in year 2017. It includes 108,948 frontal-view CXRs of 32,717 unique patients with eight different findings. The dataset is labeled report parsing (NLP) associated with each sample. The dataset can be downloaded from https://nihcc.app.box.com/v/ChestXray-NIHCC.
- Chestxray14: Wang et al. (2017) published the dataset in 2017 consisting of 112,120 CXR samples from 30,805 subjects. Dataset consists of 1, 024 × 1, 024 image resolution images collected from the National Institute of Health (NIH), US. It contains labels for the 14 findings, automatically generated from the reports using NLP. The dataset is publicly available and can be downloaded from https://www.kaggle.com/nih-chest-xrays/data.
- RSNA-Pneumonia: It's the dataset generated from the samples ChestXray14 dataset for pneumonia detection. It contains a total of 30,000 CXRs with pneumonia annotations with a 1, 024 × 1, 024 resolution. The annotations include lung opacities, resulting in samples with three classes normal, lung opacity, and not normal (Stein et al., 2018). The dataset can be downloaded from https://www.kaggle.com/c/rsna-pneumonia-detection-challenge/data.
- Ped-Pneumonia: Kermany et al. (2018a) published the dataset in 2018, consisting of 5856 pediatric CXRs. The data is collected from Guangzhou Women and Children's Medical Center, Guangzhou, China. The dataset is labeled as viral and bacterial pneumonia. It also contains samples as normal. The dataset can be downloaded from https://data.mendeley.com/datasets/rscbjbr9sj/2.
- CheXpert: Irvin et al. (2019) published one of the largest chest X-ray datasets consisting of 224,316 images with a total of 65,240 subjects in the year 2017. It took the authors almost 15 years to collect the dataset from Stanford Hospital, US. The dataset contains labels as presence, absence, uncertainty, and no mention of 12 abnormalities, no findings, and the existence of support devices. All these labels are generated automatically from radiology reports using a rule-based labeler (NLP). The dataset can be downloaded from https://stanfordmlgroup.github.io/competitions/chexpert/.
- CXR14-Rad-Labels: This (2020) introduced the dataset as the subset of the ChestXray14, consisting of 4 labels for 4,374 studies and 1,709 subjects. The annotations are provided by the cohort of radiologists and are made available along with agreement labels.
- MIMIC-CXR: Johnson et al. (2019) published the dataset in the year 2019 with 371,920 CXRs collected from 64588 subjects admitted to the emergency department of Beth Israel Deaconess Medical Center. It took authors almost 5 years to collect the dataset, and it is made available in two versions; V1 and V2. V1 contains images with 8-bit grayscale images in full resolution, and V2 contains DICOM images with anonymized radiology reports. The labels are automatically generated by report parsing. The dataset can be downloaded from https://physionet.org/content/mimic-cxr/.
- SIIM-ACR: Anuar (2019) is Kaggle challenge dataset for pneumothorax detection and segmentation. It is believed by some researchers that the data samples are taken from the ChestXray14 dataset; however, no official confirmation is made about this. CXRs are available as DICOM images with 1, 024 × 1, 024 resolution.
- Padchest: Bustos et al. (2020) published the collected dataset in year 2020, consisting of 160,868 CXRs, 109,931 studies and 67,000 subjects. It took the authors almost 8 years to collect the dataset from the San Juan Hospital, Spain. The dataset is labeled using domain experts for a set of 27,593 images, and for the rest of the data, an RNN was trained to generate the labels from reports.
- BIMCV: Vayá et al. (2020) introduced the dataset for COVID-19 in year 2020. It includes of CXRs, CT scans and laboratory test results. The dataset is collected from Valencian Region Medical ImageBank (BIMCV). It consists of 3,293 CXRs from 1,305 COVID-19-positive subjects.
- COVID abnormality annotation for X-Rays (CAAXR): Mittal et al. (2022) proposed the dataset with annotations on the existing BIMCV-COVID-19+ dataset performed by the radiologists. The dataset contains annotations for different findings such as atelectasis, consolidation, pleural effusion, edema and others. CAAXR contains a total of 1,749 images with 3,943 annotations. The dataset can be downloaded from https://osf.io/b35xu/ and http://covbase4all.igib.res.in/.
- COVIDDSL: The dataset was released in 2020 for COVID-19 detection (Hospitales, 2020). The dataset is collected from the HM Hospitales group in Spain and includes CXRs from 1725 subjects along with detailed results from laboratory testing, vital signs etc.
- COVIDGR: Tabik et al. (2020) released the dataset, collected from Hospital Universitario Clínico San Cecilio, Granada, Spain. It consists of 852 PA CXRs, with labels for positive and negative COVID-19. The dataset also includes the severity of COVID-19 in positive cases.
- COVID-CXR: Cohen et al. (2020) released the dataset for COVID-19 with a total of 930 CXRs. The dataset includes samples from a large variety of places. It includes data collected from different methods, including screenshots from the research papers. The dataset is labeled as the label mentioned in the source and is available in PNG or JPEG format. The dataset can be downloaded from https://github.com/ieee8023/covid-chestxray-dataset.
- VinDr-CXR: Nguyen et al. (2020) proposed the dataset collected from the two major hospitals of Vietnam from 2018 to 2020. The dataset includes 18,000 CXRs, 15,000 samples for training and 3,000 for testing. The annotations are made manually by 17 expert radiologists for 22 local labels and six global labels. The samples for the training set are labeled by three radiologists, while the testing set is labeled independently by five radiologists. Images in the dataset are available in DICOM format and can be downloaded from https://vindr.ai/datasets/cxr after signing the license agreement.
- Brax: Reis (2022) introduced the dataset which includes 40,967 CXRs, 24,959 imaging studies for 19,351 subjects, collected from the Hospital Israelita Albert Einstein, Brazil. The dataset is labeled for 14 radiological findings using report parsing (NLP). Dataset is made available in both DICOM and PNG format. The dataset can be downloaded from https://physionet.org/content/brax/1.0.0/.
- Belarus: is used in many papers and consists of 300 CXR images. However, the download link is not available and also further details about the dataset are missing as well.

**Table 7:** Illustrates the available CXR datasets in the literature.

**Name**	**Number of Images (I)/Patients (P)**	**View position**	**Global labels**	**Local labels**	**Image format**	**Labeling method**
JSRT (Shiraishi et al., 2000)	I: 247	PA: 247	3	N/A	DICOM	Radiologist
Open-i (O) (Demner-Fushman et al., 2012)	I: 7910	PA: 3955, LL: 3955	N/A	N/A	DICOM	Radiologist
NLST (Team, 2011)	I: 5493	No public information is available. The dataset was reported by Lu et al. (2019)				
Shenzhen (Jaeger et al., 2014)	I: 340	PA: 340	2	N/A	PNG	Radiologist
Montgomery (Jaeger et al., 2014)	I: 138	PA: 138	2	N/A	PNG	Radiologist
Indiana (Demner-Fushman et al., 2016)	I: 7466	PA: 3807, LL: 3659	N/A	N/A	N/A	Radiology reports
Chestxray8 (Wang et al., 2017)	I: 108K+, P: 32,717	N/A	N/A	8	PNG	Report parsing
Chestxray14 (Wang et al., 2017)	I: 112K, P: 31K	PA: 67K, AP: 45K	No	14	PNG	Report parsing
RSNA-Pneumonia (Stein et al., 2018)	I: 30K	PA: 16K, AP: 14K	1	N/A	DICOM	Radiologist
Ped-Pneumonia (Kermany et al., 2018a)	I: 5856	N/A	2	N/A	JPEG	Radiologist
CheXpert (Irvin et al., 2019)	P: 65K, I: 224K	PA: 29K, AP: 16K, LL: 32K	N/A	14	JPEG	Report parsing Cohort of Radiologists
CXR14-Rad-Labels (This, 2020)	P: 1709, I: 4374	AP: 3244, PA: 1132	4	N/A	PNG	Radiologist
MIMIC-CXR (Johnson et al., 2019)	P: 65K, I: 372K	PA+AP: 250K, LL: 122K	N/A	14	JPEG(V1) DICOM(V2)	Report Parsing
SIIM-ACR (Anuar, 2019)	I: 16K, P: 16K	PA: 11K, AP: 4799	1	N/A	DICOM	Radiologist
Padchest (Bustos et al., 2020)	P: 67K, I: 160K	PA: 96K, AP: 20K, LL: 51K	N/A	193	DICOM	Report parsing Radiologist Interpretation of reports
BIMCV (Vayá et al., 2020)	P: 9129, I: 25,554	PA: 8,748, AP: 10,469, LL: 6,337	1	N/A	PNG	Laboratory Reports
CAAXR (Mittal et al., 2022)	P: 1749, I: 1749	Not mentioned	1	N/A	PNG	Cohort of radiologists
COVIDSSL (Hospitales, 2020)	P: 1,725	Mostly AP	1	N/A	DICOM	Laboratory Reports
COVIDGR (Tabik et al., 2020)	I: 852	PA: 852	2	N/A	JPEG	Radiologist
COVID-CXR (Cohen et al., 2020)	I: 866, P: 449	PA: 344, AP: 438, LL: 84	4	N/A	JPEG	Radiologist
VinDr-CXR (Nguyen et al., 2020)	I: 18K	PA: 18K	6	22	DICOM	Radiologist
Brax (Reis, 2022)	P: 19,351, I: 40,967	Numbers are not mentioned	N/A	14	DICOM + PNG	Report parsing
Belarus (Rosenthal et al., 2017)	I: 306, P:169	No other information is available				

The table presents the description of each dataset with the number of images, patients, the available format of images, view position and labeling (annotation) method. The global label refers to the label single label assigned to the image for multiclass settings while local labels refer to multiple labels assigned to a single image for different findings present in multilabel settings. PA stands for Posterior to anterior, AP stands for Anterior to posterior and LL stands for lateral view. K refers to 1,000. N/A stands for not available.

### 4.1. Discussion

Generating large datasets in the medical domain is always a challenging process due to data privacy concerns and the need for expert annotators. While several existing datasets have enabled different research threads for CXR-based image analysis for disorders such as TB and pneumonia, the number of annotated samples in these datasets is less for modern deep learning based algorithm development. Further, local ground truth labeling plays an important role in disease classification and detection, and improves explainability. Existing datasets, in general, lack variability in terms of sensors and demographics. For many thoracic disorders, such as Pneumoconiosis, COPD, and lung cancer, there is a lack of publicly available datasets. On the other hand, datasets for the recent COVID-19 pandemic are collected from different hospitals across the globe with fewer samples and limited labels. Only a few datasets have associated local labels; for instance, Chestxray14 and CheXpert. These labels are generated using the report parsing method and results in high label noise. This may increase higher chances of missing labels due to the absence of findings in radiology reports on which the report parser (NLP algorithm) is designed. This draws the attention to carefully handling the labeling process while releasing the datasets to avoid any errors during deep model training.

## 5. Evaluation metrics

This section covers different metrics used to evaluate the proposed approach in the existing literature. Table 8 summarizes the various metrics that are used to evaluate different tasks in CXR-based image analysis.

**Table 8:** Summarizes the metrics used for assessing the performance of different tasks performed by an ML/DL model.

**Image enhancement**	**PSNR**		**SSIM**		**MSE**		**MAXERR**		**L2rat**
Segmentation	Intersection over Union (IOU)			Dice Coefficient		Pixel accuracy	
Classification	Sensitivity	Specificity	Accuracy	Precision		F1-score		AUC-ROC Curve	
Fairness	Demographic Parity	Equalized odds	Degree of bias	Disparate impact	Predictive Rate Parity	Equal opportunity	Treatment Equality	Individual Fairness	Counterfactual fairness
Image captioning	BLEU		METEOR		ROGUE-L		CIDEr	SPICE	

### 5.1. Image enhancement task

To assess the quality of images for different enhancement techniques, the difference between the original and enhanced image is calculated using the following metrics.

- Peak signal to noise ratio (PSNR): It is a quality assessment metric and is expressed as the ratio of the maximum possible power of the original signal to the power of the noisy signal.
- Structural Similarity Index (SSIM): It is a quality measure used to compare the similarity between two images.
- Mean squared error (MSE): It is a quality assessment measure and is defined as the accumulative sum of square error between enhanced and original images.
- MAXERR: It is the maximum absolute squared error of the specified enhanced image with a size equal to that of the original image (Huynh-Thu and Ghanbari, 2008).
- L2rat: It is defined as the squared norm of the enhanced image to the original image (Huynh-Thu and Ghanbari, 2008).

### 5.2. Segmentation task

Segmentation approaches aim to find the ROI in a given image. In order to evaluate the segmentation algorithms for generating the prediction mask, and compare that with the ground truth mask, the following performance metrics are used :

- Intersection over Union (IOU): It is also called as Jaccard Index. It is defined as the ratio of intersection over the union of area for the predicted mask to the area of the ground truth mask. The IOU value lies between 0 for poor overlap and 1 for complete overlap. Values above 0.5 are considered decent for the algorithm. It is defined as;

$$IOU=\frac{predicted\;mask\;area\cap groundtruth\;mask\;area}{predicted\;mask\;area\cup groundtruth\;mask\;area}$$

- Dice Coefficient: It is also defined as an F1 score. It is defined as the ratio of twice the area of overlap between the predicted mask and ground truth mask to the total number of pixels for both masks. It is similar to the IOU. Mathematically, it is defined as

$$DiceCoefficient=\frac{\left(2*Area\;of\;overlap\right)}{sum\;of\;pixels\;combined}$$

- Pixel accuracy: It is another metric for evaluating semantic segmentation. It is defined as the percentage of pixels that are correctly classified. It can give misleading results for the minor class. Mathematically, it is defined as the ratio of correctly classified pixels to the sum of all the pixels. For a binary image, it is defined as;

$$\begin{array}{l} PixelAccuracy=\\ \frac{True\;Positive+True\;Negative}{True\;Positive+True\;Negative+False\;Positive+False\;Negative} \end{array}$$

### 5.3. Classification task

To evaluate the ML model for the classification task, the following metrics are widely used in the literature.

- Sensitivity: aka recall, is the proportion of the actual positive samples that are correctly identified as positive. It indicates what percent of actual disease affected patients were detected by the model. Mathematically, it is defined as:

$$\begin{array}{l} Sensitivity(Recall)=\frac{True\;Positives}{True\;Positives+False\;Negatives}\; \end{array}$$

- Specificity: aka true negative rate, refers to the fraction of the samples' actual negative cases from all the predicted negative cases. It indicates what percent of the disease-negative patients are detected as positive (False positive) Mathematically, It can be defined as:

$$\begin{array}{l} Specificity(True\;Negative\;Rate)=\\ \frac{True\;Negatives}{True\;Negatives+False\;Positives} \end{array}$$

- Accuracy: It is defined as the number of correctly classified samples from the total number of samples. It shows often the model predicts the class labels accurately. However, it can be misleading sometimes, and class wise accuracy is preferred over overall accuracy.

$$\begin{array}{l} Accuracy=\\ \frac{True\;Positives+True\;Negative}{True\;Positives+False\;Negatives+True\;Negative+False\;Positive} \end{array}$$

- Precision: Also known as a positive predictive value, is the ratio of positive samples that are accurately predicted. It emphasizes how many correctly predicted samples are actually TB positive. It is majorly used in cases where false positive are of more importance than false negatives. Mathematically it is defined as:

$$Precision=\frac{True\;Positive}{True\;Positive+False\;Positive}$$

- F1-score: It is defined as the harmonic mean of precision and recall. It reaches the maximum value when both precision and recall are equal. It is of high use in cases where both false positives and true negatives are of equal concern. Mathematically, it is defined as

$$F1-score=2*\frac{Precision*recall}{Precision\;+Recall}$$

- AUC-ROC Curve: It tells the probability of separating samples of negative class from positive class samples based on different thresholds. For different thresholds, a plot is obtained for different values of True Positive Rate (TPR) and their corresponding False Positive Rate (FPR) values. For example, it is not always necessary to have a particular threshold such as 0.5 and classify a patient as a positive for disease if value is >0.5 and negative if value is <0.5. A set of different thresholds is used to find an optimal threshold, where both positive and negative patients are classified best by the model.

$$TPR=Sensitivity=\frac{True\;Positive}{True\;Positive+False\;Negative}$$

$$FPR=1-Specificity=\frac{False\;Positive}{False\;Positive+True\;Negative}$$

### 5.4. Fairness metrics

DL models are black boxes and act differently across protected attributes such as age, gender, race, or socio-economic status. Fair or bias-free decisions show zero affinity of the model toward any individual or subgroup in the population set based on any inherent characteristics. To evaluate a deep model for exhibiting disparities across subgroups, fairness metrics demonstrate whether the decisions are fair or not for the protected attributes. These allow us to avoid any ill-treatment toward any subgroup after the deployment of the model in real-world settings.

To assess the model performance for different protected attributes in the population, the following are a few fairness metrics used in the literature for measuring bias or assessing the fairness of AI Systems.

- Demographic parity: It is defined as the probability of being classified with the favorable label and is independent of group membership (protected and unprotected). It is also known as Statistical Parity (Zafar et al., 2017). For a disease classification problem, demographic parity is witnessed if the samples are not equally classified independent of the membership of being male or female.
- Equalized odds: It is defined as both false-positive and true-positive rates for protected and unprotected groups being the same. It is also known as Separation, Positive Rate Parity (Zafar et al., 2017). For a For a disease classification problem, if training data patients and are males only and all females as normal samples and equalized odds is satisfied if the model equally classifies or misclassifies the positive samples irrespective of whether that's male or female at the test time.
- Degree of bias: It is defined as the standard deviation of classification accuracy across different subgroups of a demographic group.
- Disparate impact: It is defined as the ratio of probabilities of being classified with the favorable label between protected and unprotected groups close to one. For instance, for a disease classification problem, if the model is favoring males over females and thus showing disparate impact.
- Predictive rate parity: It is defined as the fraction of correct positive predictions that is the same for protected and unprotected groups (Chouldechova, 2017). For example, the predictive parity rate for the disease classification is achieved if the precision for both subgroups (e.g., male and female) is the same. Predictive rate parity is also known as predictive parity.
- Equal opportunity: It is defined as the true positive rate being the same between protected and unprotected groups (Hardt et al., 2016). For example, for a disease classification problem, if disease-positive patients are only males and all females as normal samples. Equal opportunity is achieved if the model still predicts samples equally irrespective of whether they are male or female (protected attributes)
- Treatment equality: It is defined if both protected and unprotected groups have an equal ratio of false negatives, and false positives (Berk et al., 2021).
- Individual fairness: It is defined as the metric which treats similar individuals similarly (Dwork et al., 2012). For instance, Individual fairness is satisfied if samples from two different individuals with the same severity for a disease are equally treated by the model for disease classification.
- Counterfactual fairness: It considers a model to be fair for a particular individual or group if its prediction in the real world is the same as that in the counterfactual world where the individual(s) had belonged to a different demographic group. It provides a way to check the possible way to interpret the causes of bias and the impact of replacing only the sensitive attributes (Russell et al., 2017).

### 5.5. Report generation

To evaluate the report/caption generation for images, the following are the widely used evaluation metrics. All these metrics find the similarity (n-gram matching) solely between the ground truth and predicted captions without taking the image into consideration.

- BLEU: Bilingual Evaluation Understudy measures the quality of the translated sentences with reference to the similarity between predicted and labels caption, based on the *n-gram* matching rule. Its value lies between 0 and 1 (Papineni et al., 2002). It is based on the n-gram co-occurrence frequency between the reference and predicted captions.
- METEOR: Metric for Evaluating Translation with Explicit Ordering calculates the precision and recall and then takes a harmonic mean for the query image caption (Banerjee and Lavie, 2005). Unlike BLEU, it measures the word-to-word matching and calculates recall for accurate word matching.
- ROGUE-L: Recall-oriented Understudy for Gisting Evaluation is used to evaluate the co-occurrence of *n-tuples* in the abstracts. It is the evaluation method to calculate the machine's fluency of translation (Lin and Hovy, 2003). It uses the concept of dynamic programming to find the longest common subsequence between the reference and predicted caption and to uses it to calculate the recall to determine the similarity between the two captions. Higher the value of ROGU-L, better the model, however, it doesn't consider the grammatical accuracy or the semantic level of description.
- CIDEr: Consensus-based Image Description Evaluation calculates the similarity between the reference and predicted caption by considering each sentence as a document. The Cosine angle of the word frequency-inverse document frequency (TF-IDF) vector is calculated. The final result is obtained by averaging the similarity of tuples of different lengths (Vedantam et al., 2015).
- SPICE: Semantic Propositional Image Caption Evaluation uses the graph-based semantic representation to encode the objects, attributes, and relationships in the description sentence and evaluate the description sentence at the semantic level (Anderson et al., 2016). It faces challenges with repetitive sentences, however, generates captions with a high correlation with human judgement.

## 6. Open problems

Based on the literature review, here we present the open challenges in AI-based CXR analysis that require attention from the research community.

- Unavailability of data: Due to the inaccessibility of publicly available datasets for many lung diseases such as the detection of Pneumoconoisis from CXRs, it is challenging to create large-scale models for different lung diseases. In addition, a number of datasets are from a few specific countries like the USA. In order to build generalizable models, it is important to create large-scale datasets with diversity.
- Small sample size problem and interoperability: Existing work is done on fewer in-house collected chest X-ray samples. Developing a robust and generalizable deep learning-based model requires a huge amount of training data. The datasets are very small in size compared to general object detection problems (for instance, the ImageNet dataset). Since the scanners might vary according to locations, deep models need to be aware and invariant of the dependency of learning a specific portion of the dataset, specifically for the datasets where data is collected from different hospitals.
- Multilabel and limited label problem: A given chest X-ray of the patient suffering from Pneumoconoisis or TB develops multiple manifestations such as nodules, emphysema, tissue scarring, and fibrosis, which results in multilabel problems. On top of the limited accessible data, data labeling is also a challenge and requires detailed inputs from domain experts. Chest diseases are mainly focused on the lung fields; however, ground mask to segment the CXRs is scanty in the literature. Domain experts such as chest radiologists and pulmonologists must be consulted for data annotation and labeling, and encourage collaboration with more hospitals, radiologists and pulmonologists.
- Low-quality images: The data collected may not always be of high quality. Samples also suffer alignment problems, which sometimes need to be fixed. Handling noisy data contributes to another challenge for algorithm design. A robust AI-based pipeline is needed to handle noise and image registration for lung disease detection.
- Lung disease correlation and co-occurrence: The presence of Pneumoconoisis and its related diseases, such as TB, share similar pathology, often resulting in misdiagnosis. Two diseases can be associated with the same patient, for instance, Silicotuberculosis (silicosis and TB). A similar problem is faced with pneumonia with its three variants; viral, bacterial and COVID-19.
- Trusted AI: Building trust in machine intelligence, especially for medical diagnoses, is crucial. Data bias among different demographics and sensors can result in inaccurate diagnostic decisions. Moreover, data privacy for accessing any patient data is of utmost priority. In addition, incorporating algorithmic explainability is a significant task to handle. Explainability in models can play an essential role in developing automated disease detection solutions to ease the workload in hospitals, decrease the chances of misdiagnoses, and encourage building trust in the diagnostic assistants. In particular, deep models face data bias and adversarial attacks in machine intelligence-based prediction. To harness the efficacy of deep models for automatic disease detection using CXRs, there is a need to build trustable systems with high fairness, interpretability and robustness.

## 7. Conclusion

CXR based image analysis is being used for detecting the presence of diseases such as TB, Pneumonia, Pneumoconiosis and COVID-19. This paper presents a detailed literature survey of AI-based CXR analysis tasks such as enhancement, segmentation, detection, classification, image and report generation along with different models for detecting associated diseases. We also present the summary of datasets and metrics used in the literature as well as the open problems in this domain. It is our assertion that there is a vast scope for improving automatic and efficient algorithm development for CXR-based image analysis. The advent of AI/ML techniques, particularly deep learning models, provides a scope of responsible, interpretable, privacy friendly digital assistance for thoracic disorders and addresses several open problems/challenges. Furthermore, novel CXR datasets must be prepared and released to encourage development of novel approaches for various disorders.

## Author contributions

YA conducted the literature review. YA, RS, and MV wrote the paper. All authors finalized the manuscript.

## Footnotes

^1^ https://www.healthimages.com/mri-vs-ct-scan/

^2^ https://www.nibib.nih.gov/science-education/science-topics/magnetic-resonance-imaging-mri

^3^ https://radiopaedia.org/articles/chest-radiograph

^4^ https://www.radiologymasterclass.co.uk/tutorials/chest/chest_quality/chest_xray_quality_rotation
